# Integrating MALDI-TOF Mass Spectrometry and Machine Learning for Rapid and Clinically Relevant Differentiation of MRSA and MSSA

**DOI:** 10.3390/pathogens15020191

**Published:** 2026-02-09

**Authors:** Abdurrahman Gülmez, Ayşe Nur Ceylan, Selda Kömeç, Beyza Öncel, Yasin Sağlam

**Affiliations:** 1 Medical Microbiology Laboratory, Aydin Ataturk State Hospital, Aydin 09020, Türkiye; 2 Medical Microbiology Laboratory, Istanbul Basaksehir Cam and Sakura City Hospital, Istanbul 34480, Türkiye; aysenurceylan1011@gmail.com (A.N.C.); seldakomec@gmail.com (S.K.); beyza.asker1@gmail.com (B.Ö.); 3 Vezirkopru District Directorate of Health, Samsun 55900, Türkiye; dr.yasinsaglam@gmail.com

**Keywords:** matrix assisted laser desorption ionization mass spectrometry, *Staphylococcus aureus*, methicillin-resistant *Staphylococcus aureus*, machine learning, antimicrobial drug resistance

## Abstract

Matrix-assisted laser desorption/ionization time-of-flight mass spectrometry (MALDI-TOF MS) is routinely used in clinical microbiology for rapid species identification; however, its potential to support early antimicrobial decision-making remains under active investigation. Rapid discrimination of methicillin-resistant *Staphylococcus aureus* (MRSA) and methicillin-susceptible *Staphylococcus aureus* (MSSA) at the time of identification could facilitate earlier optimization of antimicrobial therapy and infection control measures. In this study, MALDI-TOF MS spectral data were analyzed to evaluate supervised machine learning–based differentiation of MRSA and MSSA. A total of 91 *S. aureus* isolates (37 MRSA and 54 MSSA) were included, with methicillin susceptibility determined by the cefoxitin disk diffusion test according to EUCAST guidelines and used as the reference standard. MALDI-TOF MS spectra were acquired following standard on-plate extraction, subjected to quality control, and preprocessed prior to analysis. Principal component analysis demonstrated partial but consistent separation between MRSA and MSSA isolates. A Random Forest classifier was trained and validated using stratified 10-fold cross-validation, achieving an overall classification accuracy of 81.3% and a receiver operating characteristic area under the curve of 0.916. Class-specific analysis revealed high precision for MRSA (95.5%) and excellent recall for MSSA (98.1%). These findings indicate that MALDI-TOF MS combined with machine learning can provide clinically relevant information for rapid MRSA/MSSA differentiation and may serve as a cost-free decision-support approach in routine clinical microbiology workflows, complementing standard phenotypic susceptibility testing.

## 1. Introduction

Matrix-assisted laser desorption/ionization time-of-flight mass spectrometry (MALDI-TOF MS) has become a widely used tool in clinical microbiology laboratories for the rapid and accurate identification of microorganisms [1]. This technology enables the characterization of organisms based on their unique protein mass fingerprints, which are generated from ribosomal and other abundant cellular proteins and subsequently compared with reference databases for species-level identification [2].

In recent years, MALDI-TOF MS has attracted increasing attention not only as an identification tool but also as a potential method for predicting antimicrobial resistance [3]. In particular, the discrimination between methicillin-resistant *Staphylococcus aureus* (MRSA) and methicillin-susceptible *Staphylococcus aureus* (MSSA) isolates in clinically important pathogens such as *S. aureus* may yield results more quickly than conventional antibiogram and genotypic methods [4].

Numerous studies have shown that specific *m*/*z* peaks in MALDI-TOF MS spectra vary between MRSA and MSSA isolates [2,4,5,6,7]. For instance, some studies have identified peaks uniquely associated with MRSA. Despite these findings, there is currently no consistent, widely applicable method for using this information in routine clinical diagnosis [6]. However, potential differentiation of certain characteristic peaks has been found in spectra obtained from a wide range of clinical isolates, and these differences have been supported by in vitro phenotypic and genotypic data [7].

While traditional statistical methods have relied on *p*-values to identify differences, machine learning approaches have become increasingly popular in recent years for revealing distinct features in multivariate data and improving classification accuracy. Random Forest (RF), Support Vector Machine, and other supervised learning algorithms have been successfully used to predict antimicrobial resistance phenotypes from MALDI-TOF MS data. These methods have shown higher accuracy and better generalization compared to traditional analyses. Systematic studies have identified trends and strengths in this area, indicating that well-trained machine learning models can accurately predict resistance profiles from MALDI-TOF MS spectra [7,8,9,10].

Nevertheless, a standardized protocol to integrate MALDI-TOF MS and machine learning to differentiate between MRSA and MSSA in clinical practice has not yet been developed, and there is a lack of consistency across devices, data structures, and analysis methods [4].

The rapid identification of MRSA and MSSA using routine MALDI-TOF MS could enable earlier optimization of antimicrobial therapy and infection control decisions, potentially improve patient outcomes, and reduce unnecessary use of broad-spectrum agents [11].

Previous studies indicate that MALDI-TOF MS combined with machine learning can predict methicillin resistance in *S. aureus*. However, these studies often use centralized datasets and complex analyses that are impractical for routine diagnostic laboratories. While MALDI-TOF MS enables rapid species identification of *S. aureus*, it cannot distinguish MRSA from MSSA isolates, necessitating traditional phenotypic antimicrobial susceptibility testing that can take hours to days to yield results.

This study aimed to evaluate the feasibility and clinical decision-support value of supervised machine learning for differentiating MRSA from MSSA using MALDI-TOF MS spectra generated through standard diagnostic workflows. By using routinely acquired spectra without incurring additional costs, specialized instruments, or proprietary software, we sought to determine whether it is possible to extract resistance-related information during identification. This approach aims to complement rather than replace traditional phenotypic susceptibility testing.

## 2. Materials and Methods

### 2.1. Bacterial Isolates and Culture Conditions

MRSA isolates included in this study were obtained from a previously published, molecularly characterized isolate collection described by Ceylan et al. [12]. In the referenced study, the methicillin resistance of these isolates had been confirmed by polymerase chain reaction (PCR) detection of the *mecA* gene. Permission to use archived isolates and associated molecular data from the previous study was obtained prior to the initiation of the present research. For the current study, archived MRSA isolates were retrieved from storage and re-cultured exclusively for MALDI-TOF MS–based spectral analysis and machine learning modeling. No additional molecular testing was performed, and no patient-identifiable clinical data were accessed or used.

MSSA isolates were obtained from non-duplicate routine clinical specimens submitted to the microbiology laboratory of Basaksehir Cam and Sakura City Hospital. Specimen types included blood, wound swabs, respiratory samples, and other clinical materials. MSSA isolates were identified using standard microbiological methods, and no molecular testing was performed. A total of 91 non-duplicate *S. aureus* isolates (MRSA and MSSA) were included in the study.

All isolates were stored at −80 °C until further analysis and subcultured onto blood agar plates prior to MALDI-TOF MS analysis. As the study was retrospective and based on archived, anonymized laboratory isolates, patient-specific demographic data, such as age and sex, were unavailable and not analyzed.

### 2.2. Antimicrobial Susceptibility Testing (AST)

Methicillin susceptibility testing was performed for all *S. aureus* isolates using the cefoxitin (30 µg) disk diffusion method on the same day as MALDI-TOF MS analysis. A 0.5 McFarland bacterial suspension prepared from overnight cultures was inoculated onto Mueller–Hinton agar plates, cefoxitin disks were applied, and plates were incubated at 35 ± 1 °C for 18–24 h. Inhibition zone diameters were measured and interpreted according to EUCAST clinical breakpoints; isolates with cefoxitin inhibition zone diameters < 22 mm were classified as methicillin-resistant (MRSA), whereas isolates with zone diameters ≥ 22 mm were classified as methicillin-susceptible (MSSA). For the purpose of this study, isolates were phenotypically classified as MRSA or MSSA based on cefoxitin disk diffusion results, which served as the reference standard for all subsequent statistical and machine-learning analyses. In the isolate collection analyzed in this study, no *mecA*-positive but phenotypically cefoxitin-susceptible *S. aureus* isolates were observed.

### 2.3. MALDI-TOF MS Sample Preparation

MALDI-TOF MS sample preparation was performed using a standard on-plate extraction protocol. Following overnight incubation on blood agar plates, a single fresh bacterial colony was transferred onto a stainless-steel MALDI target plate (Bruker Daltonics, Bremen, Germany). After air drying, the bacterial spot was overlaid with formic acid to facilitate protein extraction and cell wall disruption. The spot was allowed to dry completely at room temperature. Subsequently, a matrix solution was applied to each spot and air-dried prior to analysis. The matrix consisted of a saturated solution of α-cyano-4-hydroxycinnamic acid (HCCA), prepared according to the manufacturer’s recommendations. All samples were prepared using identical procedures to ensure consistency and reproducibility across MRSA and MSSA isolates.

### 2.4. MALDI-TOF MS Data Acquisition

Mass spectra were acquired using a Bruker MALDI-TOF MS system operated with the manufacturer’s standard software (Bruker SMART/Biotyper environment, version 3.1). Spectra were recorded in linear positive ion mode within the standard mass range used for bacterial identification. For each isolate, spectra were generated from freshly prepared spots and used for subsequent spectral preprocessing and analysis. Only spectra yielding a Bruker Biotyper identification score ≥ 2.0 were included in the study.

### 2.5. Instrument Calibration and Quality Control

Prior to MALDI-TOF MS analysis, external calibration was performed according to the manufacturer’s recommendations using the Bruker Bacterial Test Standard (BTS), which contains a defined mixture of *Escherichia coli* proteins for mass accuracy calibration across the relevant *m*/*z* range. Calibration was verified before routine measurements to ensure optimal mass precision and spectral reproducibility. Quality control was implemented during data acquisition utilizing the Bruker Biotyper software environment (version 3.1). We visually inspected the acquired spectra for overall signal intensity, baseline stability, resolution, and peak distribution. Spectra that showed low signal intensity, poor resolution, or irregular peak patterns were excluded from further analysis. To reduce technical variability, all spectra were acquired using identical instrument settings and acquisition parameters throughout the study.

### 2.6. Spectral Preprocessing and Peak Detection

MALDI-TOF MS spectra (mzML) were imported into the Mass-Up software platform (version 1.0.14) for spectral preprocessing and analysis, which is an open-source tool based on the MALDIquant framework [13]. Quality control and preprocessing were applied to reduce technical noise and improve spectral comparability across isolates, including smoothing, baseline correction, and intensity normalization. Following preprocessing, peak detection and peak alignment were performed to generate a matched peak list dataset, enabling direct comparison of corresponding *m*/*z* values between MRSA and MSSA isolates. Datasets were labeled according to reference cefoxitin susceptibility results.

Principal component analysis (PCA) and inter-class comparative analysis were performed to identify discriminatory peaks, with statistical significance assessed using *p*-values and false discovery rate-corrected q-values. Peaks with *p* < 0.05 and q < 0.05 were considered statistically significant. Selected peaks were visually evaluated using overlay spectra. Machine learning–based classification was subsequently conducted using an RF algorithm implemented in the integrated Waikato Environment for Knowledge Analysis (WEKA) environment with stratified 10-fold cross-validation, and model performance was evaluated using standard classification metrics and confusion matrices. Phenotypically confirmed MRSA and MSSA isolates were used as reference categories for machine learning analyses, while instrument calibration and spectral quality control were ensured using the Bruker Bacterial Test Standard (BTS).

### 2.7. Statistical Analysis of Discriminatory Peaks

To identify spectral features associated with methicillin resistance, a comparative inter-class analysis was performed between the MRSA and MSSA groups using the matched peak list dataset. For each detected *m*/*z* value, the presence or absence of the peak across isolates was recorded and compared between groups using univariate statistical tests. Statistical significance was assessed by calculating *p*-values for each peak, followed by false discovery rate correction to obtain q-values. Peaks showing statistically significant differences after correction were considered candidate discriminatory features. The consistency of peak occurrence within each group was additionally evaluated to support biological relevance. Selected discriminatory peaks were subsequently visually validated using overlay spectra and included in downstream machine-learning analyses.

### 2.8. Machine Learning Classification and Model Evaluation

**Figure 1:**
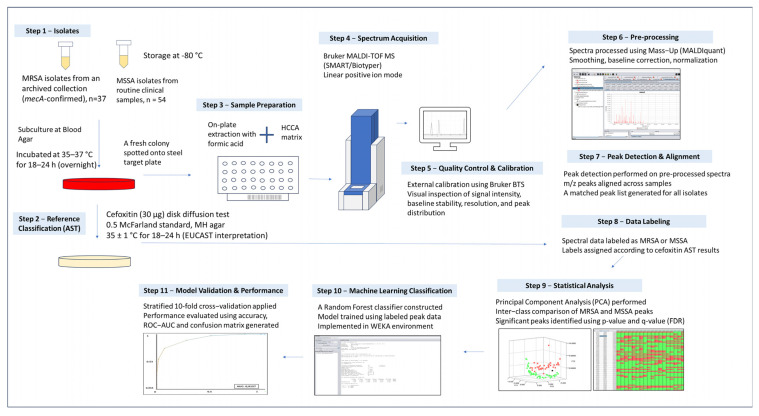
Schematic overview of the workflow used for machine learning–based discrimination of MRSA and MSSA. Clinical *Staphylococcus aureus* isolates were first subjected to routine MALDI-TOF MS identification and phenotypic antimicrobial susceptibility testing using cefoxitin disk diffusion, which served as the reference classification. MALDI-TOF MS spectra obtained during routine identification were preprocessed (baseline correction, normalization, and peak alignment) and subsequently analyzed using supervised machine learning (Random Forest). Model performance was evaluated using stratified cross-validation, and the final output included predicted MRSA/MSSA classifications and associated performance metrics.

MALDI-TOF MS spectra obtained during routine identification were used for machine learning–based discrimination of MRSA and MSSA isolates. The overall analytical workflow is summarized in Figure 1, illustrating each step from isolate processing to classification output.

Spectral data were preprocessed to improve comparability between samples, including baseline correction, normalization, and peak alignment. These steps were applied uniformly to all spectra prior to analysis. The resulting processed spectra were then used as input features for supervised machine learning.

Machine learning analyses were performed using WEKA (version 3.8), an open-source data mining and machine learning software developed by the University of Waikato, New Zealand (https://www.cs.waikato.ac.nz/ml/weka/, accessed on 1 February 2026). An RF classifier was selected, as it is a robust ensemble learning method that combines multiple decision trees to improve classification performance and reduce overfitting, particularly when handling complex, high-dimensional data such as mass spectra.

Model performance was evaluated using stratified 10-fold cross-validation, ensuring that MRSA and MSSA isolates were proportionally represented in both the training and test sets. Classification performance was assessed using standard metrics, including accuracy, sensitivity, specificity, precision, F1-score, and the area under the receiver operating characteristic curve (ROC-AUC).

The machine learning model output consisted of predicted MRSA or MSSA classifications based solely on MALDI-TOF MS spectral data, which were subsequently compared with phenotypic cefoxitin susceptibility results, used as the reference standard.

## 3. Results

### 3.1. Study Population and MALDI-TOF MS Data Overview

A total of 91 *S. aureus* isolates were included in the study, comprising 54 MSSA and 37 MRSA isolates. Isolates were obtained from a broad range of clinical specimen types, most frequently urine cultures, tracheal aspirates, nasal swabs, blood cultures, and wound/tissue specimens (Supplementary Table S1).

MALDI-TOF MS analysis was successfully performed for all isolates included in the study, and spectra with a Bruker Biotyper identification score of ≥2.0 were included in the analysis dataset. The final dataset used for statistical and machine learning analyses comprised a matched peak list containing 2663 spectral features, representing unique *m*/*z* peaks detected across all isolates after peak detection and alignment, rather than peaks derived from a single strain.

### 3.2. Global Spectral Variability Between MRSA and MSSA Isolates

**Figure 2:**
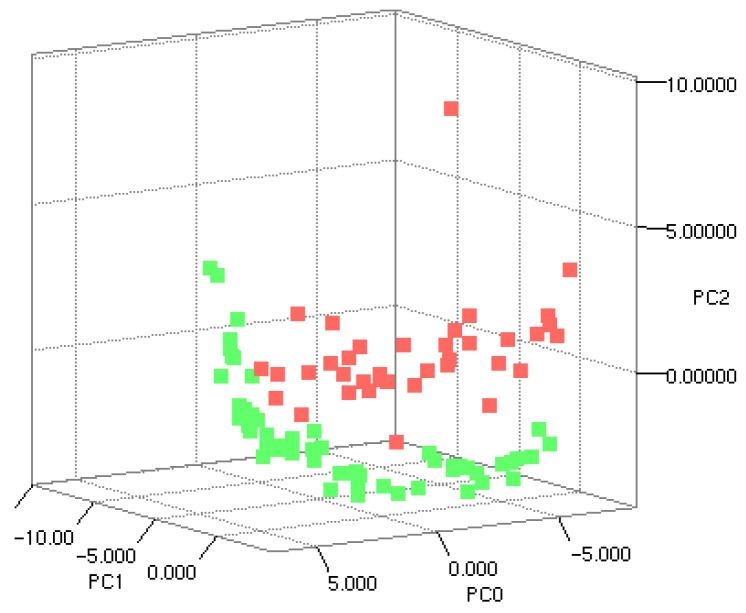
Principal component analysis (PCA) of matrix-assisted laser desorption/ionization time-of-flight mass spectrometry (MALDI-TOF MS) spectra from methicillin-resistant *Staphylococcus aureus* (MRSA) and methicillin-susceptible *Staphylococcus aureus* (MSSA) isolates. MRSA isolates are shown in red, while MSSA isolates are shown in green. Three-dimensional PCA score plot based on matched peak list data derived from MALDI-TOF MS spectra, with PC1, PC2, and PC3 representing the first three principal components. Each point represents a single *S. aureus* isolate. The plot demonstrates partial separation between the two groups, indicating underlying spectral differences associated with methicillin resistance.

Principal component analysis (PCA) was performed as an exploratory approach to visualize overall variability in MALDI-TOF MS spectra and to assess potential separation between MRSA and MSSA isolates. PCA score plots showed partial but consistent separation between the two groups, with MRSA and MSSA isolates exhibiting distinct distributions across the principal components (Figure 2). Although overlap between groups was observed, the clustering pattern suggested the presence of underlying spectral differences associated with methicillin resistance.

### 3.3. Identification of Discriminatory MALDI-TOF MS Peaks

A comparative inter-class analysis of the matched peak list dataset identified multiple *m*/*z* values that showed statistically significant differences between MRSA and MSSA isolates. After correction for multiple testing, 37 *m*/*z* values remained statistically significant based on FDR-corrected (false discovery rate) q-values and consistent group-specific presence patterns (Supplementary Table S2).

**Figure 3:**
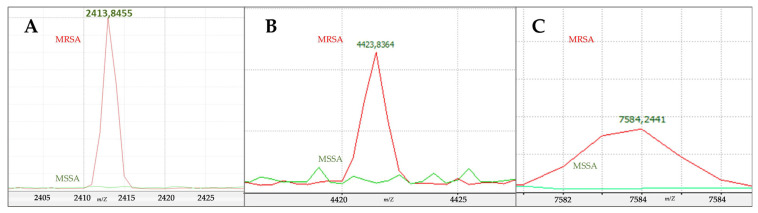
Representative overlay spectra of selected discriminatory *m*/*z* peaks. Overlay plots illustrate *m*/*z* values that showed statistically significant differences between methicillin-resistant *Staphylococcus aureus* (MRSA) and methicillin-susceptible *Staphylococcus aureus* (MSSA) isolates and exhibit consistent group-specific patterns across multiple isolates. In each panel, red lines represent MRSA spectra and green lines represent MSSA spectra. Panels (**A**–**C**) show representative overlay spectra for three different discriminatory *m*/*z* peaks, illustrating distinct intensity patterns between the two groups. These peaks are shown as representative examples; additional discriminatory peaks are reported in the main peak list (Supplementary Table S2).

To support the robustness of the statistical findings, representative discriminatory *m*/*z* values were further examined using overlay spectra. Several peaks were consistently present in the majority of MRSA isolates but absent or weak in MSSA isolates, whereas other peaks showed the opposite pattern. These reproducible patterns provided visual confirmation of the discriminatory potential of the identified peaks (Figure 3).

### 3.4. Machine Learning Classification Performance

**Figure 4:**
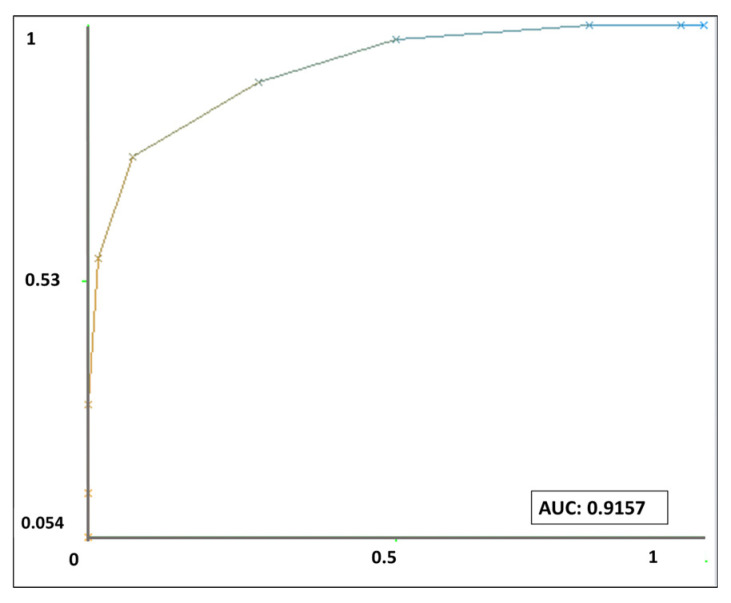
Receiver operating characteristic (ROC) curve of the Random Forest model for binary classification of methicillin-resistant (MRSA) and methicillin-susceptible (MSSA) *Staphylococcus aureus* isolates based on MALDI-TOF MS spectra. The ROC curve represents the one-vs-rest classification performance, with an area under the curve (AUC) of 0.916.

RF classification was applied to the matched-peak-list dataset to discriminate MRSA and MSSA isolates. Using stratified 10-fold cross-validation, the model achieved an overall classification accuracy of 81.3%, correctly classifying 74 of 91 isolates. The Cohen’s kappa statistic was 0.586, indicating moderate agreement beyond chance. Receiver operating characteristic (ROC) analysis demonstrated strong discriminatory performance, with an area under the curve (ROC-AUC) of 0.916 (Figure 4).

### 3.5. Class-Specific Performance and Confusion Matrix Analysis

Class-specific performance analysis revealed differences in classification performance between MRSA and MSSA isolates (Table 1). The model achieved a recall of 0.981 for MSSA isolates, indicating that nearly all were correctly classified, although precision was lower (0.768), reflecting a higher false-positive rate in susceptibility prediction. For MRSA isolates, recall (sensitivity) was 0.568, whereas precision reached 0.955, indicating that most isolates predicted as MRSA were truly resistant. The F-measure values summarize the trade-off between precision and recall for each class and highlight the model’s asymmetric classification behavior. Consistent ROC-AUC values (0.916) for both classes indicate strong overall discriminatory performance across different classification thresholds.

Analysis of the confusion matrix showed that 53 MSSA and 21 MRSA isolates were correctly classified, whereas 16 MRSA isolates were misclassified as MSSA and one MSSA isolate was misclassified as MRSA (Table 2).

## 4. Discussion

In this study, we evaluated the integration of MALDI-TOF MS–derived spectral data with RF–based machine learning to differentiate methicillin-resistant and methicillin-susceptible *S. aureus* isolates. Our findings demonstrate that MALDI-TOF MS spectra exhibit reproducible proteomic differences associated with methicillin resistance, and that these differences can be leveraged by supervised learning to provide meaningful discrimination within a clinically relevant timeframe.

Exploratory analysis using PCA revealed partial but consistent separation between MRSA and MSSA isolates, suggesting underlying spectral variability associated with resistance status. However, the observed overlap between groups confirms that unsupervised approaches alone are insufficient for reliable MRSA discrimination, a limitation that has been consistently reported in previous MALDI-TOF MS–based resistance studies [8,14,15]. These findings support the use of supervised machine learning methods to capture complex, multivariate spectral patterns that are not readily apparent through dimensionality reduction techniques.

Inter-class statistical analysis identified 37 *m*/*z* peaks that differed significantly between MRSA and MSSA isolates after FDR correction, highlighting the presence of resistance-associated proteomic signatures (*p* < 0.05, q < 0.05). Several discriminatory peaks identified in this study fall within *m*/*z* ranges previously linked to methicillin resistance. In particular, the peak at approximately *m*/*z* 2413.8 was markedly more prevalent among MRSA isolates, consistent with prior reports associating this signal with the phenol-soluble modulin mec (*PSM-mec*), a peptide encoded within certain *SCCmec* elements [2,16,17,18,19]. However, as reported in earlier studies, this peak was not universally present across MRSA isolates, reinforcing the notion that no single spectral feature can serve as a universal biomarker of methicillin resistance [5]. The variability of the *m*/*z* 2413.8 peak among MRSA isolates likely reflects regulatory control of PSM-mec by the agr system, which is frequently impaired in hospital-associated MRSA lineages. Accordingly, PSM-mec expression may be suppressed in agr-deficient strains despite mecA carriage, as previously demonstrated by Josten et al. [20].

Interestingly, several peaks were more frequently observed among MSSA isolates, such as *m*/*z* 5340.9 (75.9% in MSSA vs. 9.3% in MRSA; *p* < 0.001), suggesting that methicillin resistance–associated proteomic changes may involve not only the acquisition of resistance-linked peptides but also alterations in the expression of core cytoplasmic proteins. This observation aligns with prior studies that emphasize that methicillin resistance reflects a complex remodeling of the *S. aureus* proteome rather than simple on–off expression of a single resistance determinant [2,10,21].

By utilizing the complete matched peak list dataset (2663 spectral features) in an RF model without preliminary feature selection, leveraging the inherent ability of RF to handle high-dimensional data and rank feature importance, we achieved an overall classification accuracy of 81.3% with clinically relevant discriminatory performance, as reflected by a ROC-AUC value (0.916). This approach leverages the inherent capacity of RF algorithms to handle high-dimensional data and assess feature importance without extensive preprocessing. While large-scale studies such as that of Yu et al. (2022) primarily aimed to optimize predictive performance using centralized, multi-institutional datasets and analytically complex pipelines, the present study was designed to address a complementary question: whether robust MRSA/MSSA discrimination can be achieved using spectra generated under routine diagnostic workflows in a single laboratory setting, without additional instrumentation, proprietary software, or extensive feature engineering [7]. The observed performance is consistent with previous reports employing supervised machine learning for MRSA prediction from MALDI-TOF MS data. Although direct cross-study comparisons are limited by differences in acquisition platforms, preprocessing strategies, and isolate cohorts, reported sensitivities in RF-based MRSA models typically range from 68% to 92%, with AUC values between 0.82 and 0.88 [10,14,22]. Notably, the achieved accuracy of 81.3% and ROC-AUC of 0.916 are consistent with established benchmarks reported in the literature and indicate that meaningful discriminatory information can be extracted from routinely acquired MALDI-TOF MS spectra even without targeted feature engineering [9,14,22,23].

Class-specific performance analysis revealed a clinically significant asymmetry in the model’s predictive capabilities. We achieved very high precision for MRSA (95.5%) and an exceptional recall for MSSA (98.1%). This performance profile mirrors observations in prior studies where high specificity is often attained at the expense of sensitivity [4]. This asymmetry is likely rooted in the biological heterogeneity of MRSA lineages; the variable expression of resistance-associated proteins can lead some resistant isolates to appear spectrally similar to MSSA, resulting in the moderate sensitivity (56.8%) observed here [6].

However, from a clinical perspective, this performance profile is particularly advantageous. Because MALDI-TOF MS is already the standard method for species identification, the simultaneous extraction of resistance-related information provides actionable insights hours before conventional phenotypic susceptibility results become available, without additional costs or workflow complexity. The high precision observed for MRSA classification (95.5%) enables confident rule-in decisions, such as prompt patient isolation and initiation of appropriate empirical therapy. Conversely, the excellent MSSA recall (98.1%) is highly relevant to antimicrobial stewardship, as it may facilitate early de-escalation from empirical anti-MRSA therapy, including vancomycin, to narrower-spectrum β-lactam agents. This strategy is consistent with recommendations from the Infectious Diseases Society of America, which emphasize the superiority of β-lactams over vancomycin for MSSA infections and highlight the importance of minimizing unnecessary vancomycin exposure to reduce toxicity and the development of resistance [3,24,25]. Although the moderate sensitivity necessitates confirmation of negative predictions by standard phenotypic testing, the proposed model functions as a rapid, cost-free decision-support tool that meaningfully accelerates clinical decision-making in routine microbiology workflows.

Several limitations of this study should be acknowledged. The absence of external validation using an independent, multi-center dataset may limit the generalizability of the findings. The present study was specifically designed to evaluate MRSA/MSSA discrimination within *S. aureus* isolates. Evaluation of mecA-positive *coagulase-negative staphylococci (CoNS)* as negative or near-neighbor controls would be valuable for further assessment of specificity and generalizability; however, this was beyond the scope of the present study. Additionally, platform-specific factors and preprocessing choices may influence model performance across laboratories. As emphasized in systematic reviews, standardization of acquisition protocols, data processing pipelines, and validation strategies remains a critical challenge for the clinical implementation of machine learning–based MALDI-TOF MS applications [8].

## 5. Conclusions

In conclusion, this study demonstrates that MALDI-TOF MS spectra, when analyzed using supervised machine learning, enable reliable differentiation between MRSA and MSSA isolates. The observed performance, including high precision for MRSA and excellent recall for MSSA, supports the use of this approach as a rapid, cost-free decision-support tool within routine clinical microbiology workflows. While not intended as a standalone screening method, this strategy may facilitate early therapeutic decisions and antimicrobial de-escalation. Further multi-center validation and external testing will be required to confirm broader clinical applicability.

## Figures and Tables

**Table 1:** Class-specific performance metrics of the Random Forest classifier for methicillin-resistant *Staphylococcus aureus* (MRSA) and *methicillin-susceptible Staphylococcus aureus* (MSSA).

Class	Precision	Recall (TP Rate)	F-Measure	FP Rate	ROC-AUC
MSSA	0.768	0.981	0.862	0.432	0.916
MRSA	0.955	0.568	0.712	0.019	0.916
Weighted average	0.844	0.813	0.801	0.264	0.916

Precision: positive predictive value; Recall (TP Rate): sensitivity; F-measure: harmonic mean of precision and recall; FP Rate: false positive rate; ROC-AUC: area under the receiver operating characteristic curve.

**Table 2:** Confusion matrix of the Random Forest classifier for MRSA and MSSA discrimination.

Actual Class	Predicted MSSA	Predicted MRSA	Total
MSSA	53	1	54
MRSA	16	21	37

## Data Availability

The data supporting the findings of this study are available from the corresponding author upon reasonable request. The data are not publicly available due to ethical restrictions related to clinical bacterial isolates.

## Supplementary Materials

The following supporting information can be downloaded at: https://www.mdpi.com/article/10.3390/pathogens15020191/s1, Table S1: Distribution of MSSA and MRSA Isolates by Clinical Specimen Type, Table S2: List of statistically significant MALDI-TOF MS peaks (*p* < 0.05, FDR-corrected) distinguishing MRSA and MSSA isolates.

## Abbreviations

The following abbreviations are used in this manuscript:

MALDI-TOF MS	Matrix-Assisted Laser Desorption/Ionization Time-of-Flight Mass Spectrometry
MRSA	Methicillin-Resistant *Staphylococcus aureus*
MSSA	Methicillin-Susceptible *Staphylococcus aureus*
AST	Antimicrobial Susceptibility Testing
PCA	Principal Component Analysis
RF	Random Forest
ROC-AUC	Receiver Operating Characteristic Area Under the Curve
FDR	False Discovery Rate
EUCAST	European Committee on Antimicrobial Susceptibility Testing
