# Supplementary material for: Integrating MALDI-TOF Mass Spectrometry and Machine Learning for Rapid and Clinically Relevant Differentiation of MRSA and MSSA
Source: Pathogens. 2026 Feb 9;15(2):191. doi: 10.3390/pathogens15020191 (PMC12942793; doi:10.3390/pathogens15020191)
Supplement: Supplementary file 1 [file pathogens-15-00191-s001.zip › Supplementary Table S1.pdf]

Supplementary Table S1. Distribution of MSSA and MRSA Isolates by Clinical Specimen Type

Specimen Type	MSSA (n)	MRSA (n)	Total (n)
**Abscess culture**	5	2	7
**Sputum culture**	5	1	6
**Nasal swab culture**	7	4	11
**Urine culture**	14	9	23
**Blood culture**	7	5	12
**Ear culture**	1	1	2
**Sterile body fluid (blood culture bottle)**	1	0	1
**Tracheal aspirate culture**	5	8	13
**Wound / tissue culture**	9	7	16
**Total**	**54**	**37**	**91**
