# Supplementary material for: Integrating MALDI-TOF Mass Spectrometry and Machine Learning for Rapid and Clinically Relevant Differentiation of MRSA and MSSA
Source: Pathogens. 2026 Feb 9;15(2):191. doi: 10.3390/pathogens15020191 (PMC12942793; doi:10.3390/pathogens15020191)
Supplement: Supplementary file 1 [file pathogens-15-00191-s001.zip › Supplementary Table S2.pdf]

Supplementary Table S2. List of statistically significant MALDI-TOF MS peaks ($p < 0.05$, FDR-corrected) distinguishing MRSA and MSSA isolates

Order	peak	*p*	q-value	MSSA % (n:54)	MRSA % (n:37)
1	5340.917	0.00000048	0.0000029	75.92592593	9.375
2	4824.0146	0.00000057	0.00063092	0	40.625
3	9611.6191	0.00000071	0.00063092	42.59259259	0
4	3285.3809	0.00000013	0.00087017	3.703703704	43.75
5	7168.9668	0.00000016	0.00087078	70.37037037	21.875
6	4829.938	0.00000044	0.001977884	38.88888889	0
7	6391.5005	0.00000053	0.002023296	37.03703704	0
8	9876.9824	0.0000007	0.00236041	48.14814815	6.25
9	7584.2441	0.00000095	0.00283378	22.22222222	75
10	6778.6465	0.00000011	0.002869058	51.85185185	9.375
11	10474.76	0.00000012	0.002869058	27.77777778	84.375
12	10478.19	0.00000012	0.002869058	72.22222222	15.625
13	3792.2554	0.00000018	0.003435171	0	34.375
14	4423.8364	0.00000018	0.003435171	0	31.25
15	3583.9485	0.00000025	0.004602248	77.77777778	31.25
16	6701.6807	0.00000043	0.007283883	18.51851852	59.375
17	5770.4111	0.00000047	0.00737864	51.85185185	6.25
18	5670.2847	0.00000073	0.010909833	5.555555556	46.875
19	5070.5674	0.0000008	0.011268483	42.59259259	3.125
20	2413.8455	0.0000001	0.014322338	18.51851852	68.75
21	9662.3271	0.00000013	0.017006209	53.7037037	15.625
22	14471.98	0.00000015	0.019134214	0	28.125
23	8928.042	0.00000016	0.019134214	51.85185185	12.5
24	13549.27	0.00000017	0.019134214	22.22222222	59.375
25	13554.46	0.00000023	0.024372688	72.22222222	34.375
26	13772.44	0.00000023	0.024372688	27.77777778	68.75
27	6926.1421	0.00000025	0.025479136	57.40740741	21.875
28	8890.9277	0.0000003	0.028772933	79.62962963	31.25
29	8408.2637	0.00000033	0.030903856	50	12.5
30	15154.66	0.00000038	0.034175426	53.7037037	12.5
31	4702.2593	0.00000041	0.034551393	1.851851852	25
32	2197.2725	0.00000044	0.034551393	31.48148148	3.125
33	3798.707	0.00000044	0.034551393	31.48148148	3.125
34	2908.9233	0.00000045	0.034551393	0	25
35	6235.5142	0.00000045	0.034551393	0	21.875
36	10411.72	0.00000051	0.038259589	55.55555556	12.5
37	11130.23	0.00000065	0.046913308	12.96296296	53.125

**p*-values were calculated using Fisher's exact test; q-values were adjusted for multiple testing using the Benjamini–Hochberg false discovery rate (FDR) correction.
